# The Effect of Long-Term Duration Renal Replacement Therapy on Outcomes of Critically Ill Patients with Acute Kidney Injury: A Retrospective Cohort Study

**DOI:** 10.1155/2021/6623667

**Published:** 2021-08-30

**Authors:** Mengmeng Yang, Yun Li, Peiyao Li, Yong Fan, Yu Zhang, Rui Yuan, Lu Wang, Yan Zhao, Xiaoli Liu, Zhengbo Zhang, Hongjun Kang

**Affiliations:** ^1^Department of Critical Care Medicine, The First Medical Centre, Chinese PLA General Hospital, Beijing 100853, China; ^2^AI Lab, Global Health Drug Discovery Institute, Beijing 100084, China; ^3^Center for Artificial Intelligence in Medicine, Chinese PLA General Hospital, Beijing 100853, China; ^4^School of Biological Science and Medical Engineering, Beihang University, Beijing 100191, China

## Abstract

**Background:**

Renal replacement therapy (RRT), as a cornerstone of supportive treatment, has long been performed in critically ill patients with acute kidney injury (AKI). However, the majority of studies may have neglected the effect of the duration of RRT  on the outcome of AKI patients. This paper is aiming to explore the effect of the long duration of RRT  on the outcome of critically ill patients with AKI.

**Methods:**

This retrospective study was conducted by using the Multiparameter Intelligent Monitoring in Intensive Care II (MIMIC-II) database. The primary outcome measure of this study was the mortality at 28 days, 60 days, and 90 days in the long-duration RRT group and the non-long-duration RRT group. The secondary outcomes assessed the difference in clinical outcome in these two groups. Lastly, the effect of the duration of RRT on mortality in AKI patients was determined as the third outcome.

**Results:**

We selected 1,020 patients in total who received RRT according to the MIMIC-II database. According to the inclusion and exclusion criteria, we finally selected 506 patients with AKI: 286 AKI patients in the non-long-duration RRT group and 220 in the long-duration RRT group. After 28 days, there was a significant difference in all-cause mortality between the long-duration RRT group and the non-long-duration RRT group (*P*=0.001). However, the difference disappeared after 60 days and 90 days (*P*=0.803 and *P*=0.925, respectively). The length of ICU stay, length of hospital stay, and duration of mechanical ventilation were significantly longer in the long-duration RRT group than those in the non-long-duration RRT group. Considering 28-day mortality, the longer duration of RRT was shown to be a protective factor (HR = 0.995, 95% CI 0.993–0.997, *P* < 0.0001), while 60-day and 90-day mortality were not correlated with improved protection.

**Conclusions:**

The long duration of RRT can improve the short-term prognosis of AKI patients, but it does not affect the long-term prognosis of these patients. Prognosis is determined by the severity of the illness itself. This suggests that RRT can protect AKI patients through the most critical time; however, the final outcome cannot be altered.

## 1. Introduction

Acute kidney injury (AKI), for which morbidity and mortality have increased continually, is a ubiquitous complication in patients admitted to the intensive care unit (ICU) [1]. Renal replacement therapy (RRT), as a cornerstone of supportive treatment, has long been performed in critically ill patients with AKI [2]. Observational studies showed that this important intervention is associated with better outcomes in AKI patients [3, 4]. There are four hot topics in RRT research, including the optimal form of its modality, hemofiltration rate, and initiated time as well as the most appropriate anticoagulation method during the course of RRT [5]. Most importantly, studies found that the first three topics are associated with better outcomes in patients. Although all of the above issues remain controversial, adverse effects, such as hypotension and electrolyte disturbances, are not uncommon in the process of RRT [6]. In addition, the nutrient loss and the relative deficiency of antibiotic doses are inevitable phenomena in critically ill patients receiving RRT [7, 8]. With the duration of RRT prolonged, its side effects manifest more obviously in these patients [9]. However, a majority of studies might have neglected the effect of the duration of RRT on the outcome of AKI patients. Studies regarding the relationship between long-duration RRT and the outcome of critically ill patients are scarce. A study in critically ill children showed that the survival rate of long-duration RRT was similar to that of shorter-duration RRT [10]. In contrast, long-duration RRT in adult AKI patients is correlated with higher mortality [11]. In these reports, the time span of “long duration” is inconsistent, the duration of RRT is calculated ambiguously, and the sample sizes are relatively small, all of which may affect the accuracy of the results.

In this study, we used a precise time interval to define the “long-duration of RRT,” aiming to explore the effect of the long duration of RRT on the outcome of critically ill patients with AKI.

## 2. Materials and Methods

### 2.1. Setting

This retrospective study was conducted by using the Multiparameter Intelligent Monitoring in Intensive Care II (MIMIC-II) database, version 3.0. The MIMIC-II database (version 2.6) is a publicly available clinical database developed by the Massachusetts Institute of Technology (MIT), Phillips Healthcare, and Beth Israel Deaconess Medical Center (BIDMC). This database contains data from more than 32,000 critically ill patients who were treated in the ICUs at BIDMC from 2001 to 2008. Two types of data were collected in this database. The patient demographics, disease diagnoses, International Classification of Disease (ICD) codes, and laboratory measurement results could be extracted from the clinical data, while information such as blood pressure and heart rate could be obtained from the physiological data. The need for informed consent was waived [12]. Version 3.0 of MIMIC-II was updated based on version 2.6, now covering patients in the ICUs of BIDMC from 2001 to 2012. Over 15,000 adult patients were added to the previous database, resulting in total counts of MIMIC-II (version 3.0) of approximately 48,000 critically ill patients.

### 2.2. Inclusion and Exclusion Criteria

We selected all the patients treated with RRT from the MIMIC-II (version 3.0) database. The inclusion criteria for the present study were as follows: (1) aged ≥18 years; (2) admitted to the ICU for the first time; (3) received RRT; and (4) suffered from AKI. The exclusion criteria were as follows: (1) the duration of RRT  was less than 24 hours or (2) the diagnosis included end-stage renal disease (ESRD) or chronic kidney disease (CKD) without AKI.

### 2.3. Definition

AKI and CKD were defined according to the *International Classification of Diseases*, 9th Edition ICD-9 codes. The mean duration of RRT from all patients included in this study was 100.5 hours. Therefore, long-duration RRT was defined as the time equal to or greater than 120 hours (5 days). The mortality at 28 days, 60 days, and 90 days was calculated after RRT.

### 2.4. Study Outcomes

The primary outcome measure of this study was the mortality at 28 days, 60 days, and 90 days in the long-duration RRT group and the non-long-duration RRT group. The secondary outcomes assessed the difference in clinical outcomes in these two groups, including ICU mortality, hospital mortality, length of ICU stay, length of hospital stay, and duration of mechanical ventilation. Lastly, the effect of the duration of RRT on mortality in AKI patients was determined as the third outcome.

### 2.5. Data Collection

All adult patients who received RRT were screened in MIMIC-II (version 3.0) database. The baseline parameters, such as general demographic data and vital signs, were extracted from this database. The severity of illness within 24 hours before RRT initiation was evaluated by the Simplified Acute Physiology Score II (SAPS) and Sequential Organ Failure Assessment (SOFA) score. Major comorbidities were assessed using the Charlson comorbidity index. The highest creatinine levels of critically ill patients within the first 24 hours after admittance to the ICU and within 24 hours before RRT initiation were retrieved as well. In addition, the modes of RRT, the main diagnosis of AKI patients, and the basic disease of CKD were compared between the non-long-duration and long-duration RRT groups.

### 2.6. Statistical Analysis

Continuous variables were expressed as the mean ± SD when normally distributed or as median and interquartile ranges (IQR) when the data were skewed. In this study, several variables had missing values. Since the missing values represented a small proportion of all the values in each variable, we chose the mean fill method to manage normally distributed variables and the median fill method to deal with skewed variables. Categorical data were expressed as a number of cases or percentage. Parametric and nonparametric tests were used to compare the baseline and outcome characteristics of study groups. Continuous variables that fit for the normal distribution were compared using Student's *t*-test, while variables exhibiting a skewed distribution were compared using the Mann–Whitney *U* test. Categorical variables were compared using Pearson's chi-squared or Fisher's exact tests. The Kaplan–Meier method was utilized to analyze 28-day, 60-day, and 90-day survival rates of the long-duration and non-long-duration RRT groups using the log-rank test. Whether the duration of RRT was a risk factor for outcome (live group and death group) was determined by Cox proportional hazard regression models, where variables were selected from the univariate analysis (*P* value less than 0.1). Differences were considered significant when the *P* value associated with the two-sided test was less than 0.05. All data analysis was performed using SPSS version 19 (SPSS, Chicago, Il, USA).

## 3. Results

### 3.1. Population

**Figure 1:**
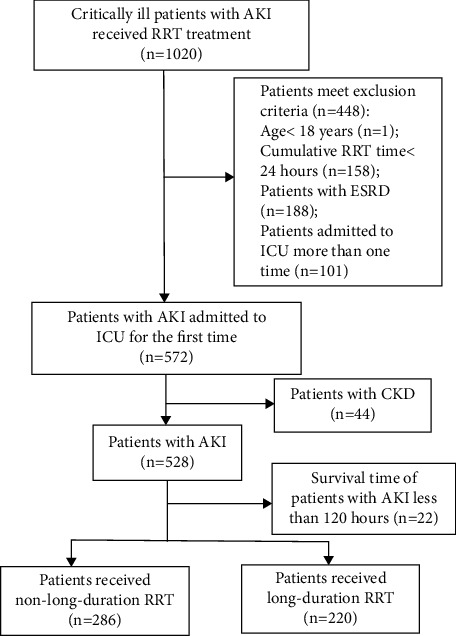
Flowchart of the study. RRT = renal replacement therapy, ESRD = end-stage renal disease, ICU = intensive care unit, CKD = chronic kidney injury, and AKI = acute kidney injury.

We selected 1020 patients in total who received RRT from the MIMIC-II database. After eliminating the patients according to the exclusion criteria, we selected 506 patients with AKI: 286 AKI patients in the non-long-duration RRT group and 220 in the long-duration RRT group. The detailed inclusion criteria and grouping method are shown in Figure 1.

### 3.2. Demographic Characteristics and Baseline Clinical Data

Baseline characteristics, stratified by the duration of RRT, are presented in Table 1. There were no differences in the demographic characteristics and vital signs between these two groups. Apparently, the creatinine level after admittance to the ICU and within 24 hours before RRT initiation was not significantly different between the two groups. However, when calculated according to the time spot, the latter was higher than the former in both the non-long-duration RRT group (4.2 (2.9, 5.7) versus 2.9 (1.9, 4.4), *P* < 0.001) and the long-duration RRT group (4.1 (3, 5.3) versus 2.8 (1.6, 4.4), *P* < 0.001). This finding implies that the renal function of AKI patients in our study declined from the time of ICU admission to RRT initiation. In the non-long-duration RRT group, more patients tended to choose the CVVHD (continuous venovenous hemodialysis) mode (*P*=0.001), whereas the CVVH (continuous venovenous hemofiltration)  + CVVHD mode was favored by the long-duration RRT group (*P* < 0.001). As determined by the SOFA score, the severity of the disease was serious in the long-duration RRT group as well (*P*=0.006). In contrast, the SAPS II score displayed no difference between the two groups (*P*=0.341).

### 3.3. Primary Outcome

**Figure 2:**
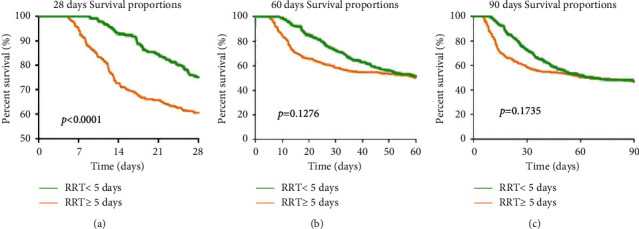
Kaplan–Meier survival curve for patients received different cumulative duration of RRT. (a) 28-day, (b) 60-day, and (c) 90-day survival analyses show that patients who received long-duration RRT have higher survival rates.

As shown in Table 2, there was a significant difference in all-cause mortality after 28 days between the long-duration RRT group and the non-long-duration RRT group (*P*=0.001). However, the difference in all-cause mortality between the groups disappeared after 60 days and 90 days (*P*=0.803 and *P*=0.925, respectively). It should be noted that the compared methods in Table 2 ignored the influence of time on AKI patients' prognosis. Considering the effect of time, Kaplan–Meier survival analysis was performed, and the results are displayed in Figure 2. The 28-day survival rate differed between the long-duration RRT group and the non-long-duration RRT group. The result of the survival analysis suggests that AKI patients who received long-duration RRT had lower mortality compared with patients in the non-long-duration RRT group (*P* < 0.001). However, the survival rates of AKI patients after 60 days and 90 days showed no significant difference between the two groups (*P*=0.1276 and *P*=0.1735, respectively). The AKI patients' median survival time 90 days after the initial RRT in this study was 64.98 days, while the median survival time of AKI patients in the non-long-duration RRT group and the long-duration RRT group was 64.36 days and 67.12 days, respectively. Among the three time points, only the 90-day mortality of AKI patients was higher than 50%.

### 3.4. Secondary Outcomes

The ICU mortality and hospital mortality were less than 50%, exhibiting no difference between the two groups. However, the length of ICU stay, length of hospital stay, and duration of mechanical ventilation were significantly longer in the long-duration RRT group than those in the non-long-duration RRT group (Table 2). This finding demonstrates that AKI patients in the long-duration RRT group require more hours of advanced life support than those in the non-long-duration RRT group.

### 3.5. Third Outcome

To explore whether the duration of RRT is associated with the outcome of AKI patients, risk factors that affect mortality in AKI patients were analyzed after 28 days, 60 days, and 90 days (Tables 3–5). The single factor analysis indicated that gender, mean arterial pressure, duration of RRT, sequential organ failure assessment, and Charlson comorbidity index were potential risk factors for mortality in AKI patients after 28 days. Cox proportional hazard models also confirmed that all 5 risk factors mentioned above are associated with patient outcome. The statistical results indicated that female (HR = 2.103, 95% CI 1.513–2.924, *P* < 0.001), higher sequential organ failure assessment (HR = 1.062, 95% CI 1.023–1.103, *P*=0.002), and Charlson comorbidity index (HR = 1.11, 95% CI 1.043–1.181, *P*=0.001) are adverse factors to outcome. Moreover, a higher mean arterial pressure and longer duration of RRT were shown to be protective factors (HR = 0.985, 95% CI 0.975–0.994, *P*=0.002 and HR = 0.995, 95% CI 0.993–0.997, *P* < 0.001, respectively). Although the age, mean arterial pressure, sequential organ failure assessment, RRT modes, and Charlson comorbidity index were risk factors for 60-day mortality, the statistical analysis indicated that mean arterial pressure (HR = 0.985, 95% CI 0.977–0.994, *P*=0.001), sequential organ failure assessment (HR = 1.044, 95% CI 1.011–1.079, *P*=0.009), and Charlson comorbidity index (HR = 1.062, 95% CI 1.001–1.126, *P*=0.048) had effect on prognosis in AKI patients. Both mean arterial pressure and Charlson comorbidity index were independent risk factors of 90-day mortality (HR = 0.988, 95% CI 0.979–0.997, *P*=0.007 and HR = 1.071, 95% CI 1.012–1.133, *P*=0.018, respectively). The effect of duration of RRT on 28-day, 60-day, and 90-day outcome by Cox proportional hazard models is shown in Table 6.

## 4. Discussion

In this retrospective study, the heart rate and SOFA score were higher in the long-duration RRT group than those in the non-long-duration RRT group, which indicates that these patients have a serious condition. As a form of RRT, CVVHD is more favored by patients with non-long-duration RRT than by patients with long-duration RRT. CVVHD is characterized by the countercurrent/cocurrent dialysate flow rate into the dialysate compartment of the hemodialyzer. The main mechanism of transmembrane solute transport is diffusion [13], which is effective mainly for the removal of small solutes [14]. CVVH relies on convection and was developed originally as an alternative for hemodynamically unstable AKI patients who could not tolerate conventional hemodialysis [15]. Due to the limited ability to control the volume by patients, CVVHD tends to be introduced in short-duration RRT [16]. Accordingly, we found that more patients received CVVHD treatment in the non-long-duration RRT group in our study. In contrast, 26.8% of patients in the long-duration RRT group received CVVHD but were transferred to CVVH, and this rate is higher than that in the non-long-duration RRT group (*P* < 0.001). This finding implies that the condition of patients in the long-duration RRT group was worse. These results were reflected by the difference in SOFA scores between the two groups (*P*=0.001), where the scores in the long-duration RRT group were higher than those in the non-long-duration RRT group.

In the present clinical study, we observed an interesting phenomenon. The survival analysis at 28 days showed that patients can substantially benefit from long-duration RRT even though the patients' conditions in the long-duration RRT group were more serious. However, this advantage in the long-duration RRT group disappeared at 60 days and 90 days when mortality did not show any statistical difference between the two groups. Regardless of the effect of time, the statistical results showed that mortality between the two groups at 28 days, 60 days, and 90 days was similar according to the Kaplan–Meier survival analysis, as shown in Table 2. An elegant study on RRT reported 60% mortality at 90 days after the initiation of CRRT among 137 patients undergoing surgery [17]. In our study, the overall mortality at 28 days, 60 days, and 90 days was 33.2%, 48.4%, and 53%, respectively, and the 90-day mortality was consistent with the results of the aforementioned study. Therefore, these findings imply that the advantage of long-duration RRT in patients' long-term prognosis is ambiguous and that this treatment method did not show any advantage in terms of ICU mortality and hospital mortality. In addition, long-duration RRT extended the length of ICU stay, length of hospital stay, and duration of mechanical ventilation.

A study published recently showed that long-duration CRRT was an independent risk factor for hospital mortality in general surgical AKI patients. When the number of days of CRRT was greater than 6 days, hospital mortality was close to 100%, indicating that long-duration CRRT is futile to improve the prognosis of AKI patients. Thus, duly withholding CRRT should be considered according to patients' outcomes, as instructional advice is put forward by researchers [18]. However, the authors also noticed that the sample size in their study was relatively small, which limits the power of the study and inhibits their ability to perform more robust statistical modeling. When screening the independent risk factor for hospital mortality, they used logistic regression analysis. However, this statistical method ignored the different time points of outcome occurrence. In our study, we chose Cox proportional hazard regression models to explore the risk factors for mortality at 28 days, 60 days, and 90 days. Different from the results of the aforementioned study, we found that the long duration of RRT is a protective factor only for 28-day mortality, while it is not associated with 60-day or 90-day mortality. In addition, the SOFA score and Charlson comorbidity index, which represent the severity of disease, are risk factors for 28-day mortality. For 60-day and 90-day mortality, only the Charlson comorbidity index showed associations with mortality. Overall, our results suggest that the long duration of RRT is an effective method to help AKI patients overcome the most critical periods, although it cannot improve the long-term prognosis of AKI patients.

RRT can remove cytokines, such as interleukin (IL)-1 beta, IL-2, IL-6 and IL-8, whose molecular weights are between 30 and 40 kDa. However, some clinical studies showed that there is no difference in the concentration of plasma cytokines during RRT [19]. Therefore, the benefits of inflammatory cytokines by RRT are still unknown. A previous study identified that macrophages participate in the injury and repair process of AKI under the stimulation of macrophage migration inhibitory factor (MIF), which can release proinflammatory cytokines [20]. Previous research also showed that proinflammatory macrophages tend to turn into wound-healing macrophages with decreased MIF concentration in cancer patients [21]. RRT has the capacity for removing MIF and improving prognosis. In addition, RRT affects energy metabolism of the body by regulating metabolic adaptation. When inflammation occurs, the level of blood glucose increases to meet elevated energy requirements [22]. However, uncontrolled hyperglycemia plays an important role in immunosuppression, which is a cause of AKI. RRT can neutralize hyperglycemia and remove glucagon. Taken together, these findings suggest that RRT is an effective method to improve prognosis in AKI patients. In septic patients, the proinflammatory response usually occurs early, while the anti-inflammatory response persists longer than the proinflammatory response, leading to late death due to immunosuppression [23]. IL-10 is an important anti-inflammatory cytokine with a molecular weight of 35–40 kDa, and it falls beyond the ability of clearance of RRT [24]. This may be the reason for sustained immunosuppression in AKI patients given RRT. Therefore, this discovery successfully explained why the prognosis of AKI patients also depends on the severity of the disease itself. As our study demonstrated, 28-day mortality is not only correlated with the duration of RRT but also affected by SOFA and Charlson scores. However, the filtration effect of RRT led to significant losses of glucose, amino acids, low-molecular-weight proteins, trace elements, and water-soluble vitamins; in particular, the duration of RRT can dramatically affect the plasma level of these nutrient substances [7]. With prolonged RRT, massive trace element loss can cause a patient to be immunocompromised and worsen the patient's condition. Hence, the loss of trace elements is currently considered a risk factor for prognosis in patients [9]. Changes in antibiotic concentration and the occurrence of complications such as hemorrhage or catheter-related infection all increased with the long duration of RRT. A new study showed that adverse cardiovascular events were increased in RRT patients, while this method did not change the prognosis of AKI patients [25]. Patients' long-term outcomes were determined by the severity of illness [4]. Our study has also suggested that prolonged RRT does not improve the long-term prognosis of AKI patients (60-day and 90-day mortality). Only basic complications of patients were shown to affect the long-term outcome, which was measured by Charlson score.

However, this cohort study also has several limitations. First, it was a single-center, retrospective analysis; thus, some unmeasured confounders cannot be excluded. Second, our study did not include the timing of RRT initiation. Violo and De Francesco [26] indicated this as a confounder that can affect the prognosis of AKI patients because the ELAIN study showed that 90-day mortality was reduced in patients who received early RRT [27]. Another excellent study found that there was no difference with regard to 90-day mortality between an early and a delayed strategy for the initiation of renal replacement therapy [1]. The timing of RRT initiation has been a focus on which many researchers debate. The latest study proved that early initiation of RRT had no association with the improvement of mortality in AKI patients [28]. Currently, we do not have the capacity to extract the data for the timing of RRT initiation, which may be correlated with the prognosis of AKI patients. Instead, the aim of our study was to explore the relationship between prolonged RRT and prognosis.

## 5. Conclusions

In our study, we found that a long duration of RRT improved short-term prognosis in AKI patients but did not change long-term prognosis. Prognosis is determined by the severity of the illness itself. This suggests that RRT is a useful method to protect AKI patients through the most critical time, while the final outcome cannot be altered.

## Figures and Tables

**Table 1:** Baseline characteristics for critically ill patients receiving long-duration and non-long-duration renal replacement therapy.

Variables	Total (*n* = 506)	Non-long-duration (*n* = 286)	Long-duration (*n* = 220)	*P* value
Age, years	64 (52, 74)	64 (53, 72)	63 (49, 75)	0.574
Male, *n* (%)	306 (60.5%)	176 (61.5%)	130 (59%)	0.577
Heart rate, beats/min	106 (90, 120)	103 (89, 119)	108 (91, 125)	0.041
Mean arterial pressure, mmHg	55 (49, 63)	55 (48, 63)	56 (50, 63)	0.526
Respiratory rate, breath/min	28 (24, 34)	28 (24, 33)	29 (24, 34)	0.445
Creatinine admitted to ICU, mg/dL	2.9 (1.8, 4.4)	2.9 (1.9, 4.3)	2.8 (1.6, 4.4)	0.485
Creatinine in 24 hours before RRT initiation, mg/dL	4.2 (3, 5.5)	4.2 (3, 5.7)	4.1 (3, 5.3)	0.391
RRT modes, *n* (%)				
CVVH	13 (2.6%)	11 (3.8%)	3 (1.3%)	0.107
CVVHD	160 (31.6%)	109 (38.1%)	51 (23.2%)	<0.001
CVVHDF	223 (44.1%)	128 (44.8%)	95 (43.2%)	0.724
CVVH + CVVHD	88 (17.4%)	29 (10.2%)	59 (26.8%)	<0.001
CVVH + CVVHDF	12 (2.4%)	5 (1.7%)	7 (3.2%)	0.293
CVVHD + CVVHDF	8 (1.5%)	3 (1%)	5 (2.3%)	0.303
Others	1 (0.2%)	1 (0.3%)	0	1
Main diagnosis class, *n* (%)				
Cardiovascular	105 (20.8%)	64 (22.4%)	41 (18.6%)	0.304
Trauma	63 (12.4%)	39 (13.6%)	24 (10.9%)	0.365
Respiratory	20 (4%)	15 (5.3%)	5 (2.3%)	0.109
Infection	124 (24.5%)	69 (24.1%)	55 (25%)	0.821
Others	194 (38.3%)	99 (34.6%)	95 (43.2%)	0.049
Sepsis, *n* (%)	95 (18.8%)	55 (19.2%)	40 (18.2%)	0.765
Chronic kidney disease, *n* (%)	176 (34.8%)	106 (37.1%)	70 (31.8%)	0.219
Simplified Acute Physiology Score II	52 (42, 63)	51 (42, 62)	54 (44, 63)	0.04
Charlson comorbidity index	5 (3, 6)	5 (3, 6)	4 (2, 6)	0.15
Mechanical ventilation, *n* (%)	459 (90.7%)	247 (86.4%)	212 (96.4%)	<0.001

RRT = renal replacement therapy; CVVH = continuous venovenous hemofiltration; CVVHD = continuous venovenous hemodialysis; CVVHDF = continuous venovenous hemodiafiltration.

**Table 2:** Clinical outcomes for long-duration and non-long-duration renal replacement therapy.

Clinical outcomes	Total (*n* = 506)	Non-long-duration (*n* = 286)	Long-duration (*n* = 220)	*P* value
Primary outcomes, *n* (%)				
28-day all-cause mortality	168 (33.2%)	113 (39.5%)	55 (25%)	0.001
60-day all-cause mortality	247 (48.8%)	141 (52.9%)	106 (48.2%)	0.803
90-day all-cause mortality	268 (53%)	152 (53.1%)	116 (52.7%)	0.925
Secondary outcomes, *n* (%)				
ICU mortality	203 (40.1%)	113 (39.5%)	90 (40.9%)	0.75
Hospital mortality	226 (44.7%)	124 (43.4%)	102 (46.4%)	0.5
Length of ICU stay, days	15 (9, 26)	11 (7, 17)	15 (22, 34.7)	<0.001
Length of hospital stay, days	24 (14, 36)	18 (12, 28)	32 (23, 43)	<0.001
Duration of mechanical ventilation, hours	262.2 (153.9, 454.4)	194 (114, 309)	409 (265.3, 685.8)	<0.001

**Table 3:** Baseline characteristics for critically ill patients at 28-day mortality.

Variables	Total (*n* = 506)	Death (*n* = 168)	Alive (*n* = 338)	*P* value
Age, years	64 (52, 74)	66 (52, 76)	63 (50, 73)	0.056
Male, *n* (%)	306 (60.5%)	116 (69%)	190 (56.2%)	0.005
Heart rate, beats/min	106 ± 23	108 ± 23	105 ± 23	0.376
Mean arterial pressure, mmHg	55 (49, 63)	53 (46, 62)	56 (50, 63)	0.005
Respiratory rate, breath/min	28 (24, 34)	29 (24, 35)	28 (24, 33)	0.081
Creatinine admitted to ICU, mg/dL	2.9 (1.8, 4.4)	2.9 (2, 4.6)	2.7 (1.6, 4.3)	0.119
Creatinine in 24 hours before RRT initiation, mg/dL	4.2 (3, 5.5)	4.2 (3.1, 5.6)	4.1 (2.9, 5.5)	0.449
Duration of RRT, hours	105.8 (63.7, 189.8)	88.3 (53.4, 148.5)	116.8 (70.06, 217.6)	<0.001
RRT modes, *n* (%)				
CVVH	14 (2.7%)	4 (2.4%)	10 (2.9%)	0.709
CVVHD	160 (31.6%)	59 (35.1%)	101 (29.9%)	0.233
CVVHDF	223 (44.1%)	69 (41.1%)	154 (45.6%)	0.338
CVVH + CVVHD	88 (17.4%)	30 (17.8%)	58 (17.1%)	0.845
CVVH + CVVHDF	12 (2.4%)	4 (2.4%)	8 (2.4%)	0.992
CVVHD + CVVHDF	8 (1.6%)	2 (1.2%)	6 (1.8%)	1
Others	1 (0.2%)	0	1 (0.3%)	1
Main diagnosis class, *n* (%)				
Cardiovascular	105 (20.8%)	32 (19%)	73 (21.6%)	0.505
Trauma	63 (12.4%)	21 (12.5%)	42 (12.4%)	0.981
Respiratory	20 (4%)	7 (4.2%)	13 (3.8%)	0.862
Infection	124 (24.5%)	41 (24.4%)	83 (24.6%)	0.984
Others	194 (38.3%)	67 (39.9%)	127 (37.6%)	0.615
Sepsis, *n* (%)	95 (18.8%)	34 (20.2%)	61 (18%)	0.552
Chronic kidney disease, *n* (%)	176 (34.8%)	56 (33.3%)	120 (35.5%)	0.629
Simplified Acute Physiology Score II	52.8 ± 14.6	55.1 ± 14.9	51.6 ± 14.4	0.717
SOFA	10 (7, 13)	11 (8, 13)	9 (7, 12)	0.009
Charlson comorbidity index	5 (3, 6)	5 (3, 7)	4 (2, 6)	0.003
Mechanical ventilation, *n* (%)	461 (91.1%)	154 (91.7%)	307 (90.8%)	0.755

RRT = renal replacement therapy; CVVH = continuous venovenous hemofiltration; CVVHD = continuous venovenous hemodialysis; CVVHDF = continuous venovenous hemodiafiltration.

**Table 4:** Baseline characteristics for critically ill patients at 60-day mortality.

Variables	Total (*n* = 506)	Death (*n* = 247)	Live (*n* = 259)	*P* value
Age, years	64 (52, 74)	66 (54, 76)	61 (49, 70)	<0.001
Male, *n* (%)	306 (60.5%)	156 (63%)	150 (57.9%)	0.228
Heart rate, beats/min	106 ± 23	106 ± 23	106 ± 24	0.151
Mean arterial pressure, mmHg	56 (49, 63)	54 (47, 62)	57 (51, 64)	0.001
Respiratory rate, breath/min	28 (24, 34)	29 (24, 35)	28 (24, 33)	0.17
Creatinine admitted to ICU, mg/dL	2.9 (1.8, 4.4)	2.7 (1.8, 4.3)	3 (1.8, 4.5)	0.346
Creatinine in 24 hours before RRT initiation, mg/dL	4.2 (3, 5.5)	4 (2.9, 5.3)	4.3 (3.2, 5.8)	0.143
Duration of RRT, hours	105.5 (63.7, 188.3)	99 (63.7, 190)	110.3 (63.6, 186.9)	0.659
RRT modes, *n* (%)				
CVVH	14 (2.7%)	7 (2.8%)	7 (2.7%)	0.928
CVVHD	160 (31.6%)	88 (35.6%)	72 (27.8%)	0.058
CVVHDF	223 (44.1%)	90 (36.5%)	133 (51.4%)	0.001
CVVH + CVVHD	88 (17.4%)	53 (21.5%)	35 (13.5%)	0.018
CVVH + CVVHDF	12 (2.4%)	6 (2.4%)	6 (2.3%)	0.934
CVVHD + CVVHDF	8 (1.6%)	3 (1.2%)	5 (1.9%)	0.519
Others	1 (0.2%)	0	1 (0.4%)	1
Main diagnosis class, *n* (%)				
Cardiovascular	105 (20.8%)	53 (21.5%)	52 (20.1%)	0.702
Trauma	63 (12.4%)	31 (12.5%)	32 (12.4%)	0.947
Respiratory	20 (4%)	9 (3.6%)	11 (4.2%)	0.728
Infection	124 (24.5%)	61 (24.7%)	63 (24.3%)	0.923
Others	194 (38.3%)	93 (37.7%)	101 (39%)	0.756
Sepsis, *n* (%)	95 (18.8%)	48 (19.4%)	47 (18.1%)	0.711
Chronic kidney disease, *n* (%)	176 (34.8%)	84 (34%)	92 (35.5%)	0.721
Simplified Acute Physiology Score II	52.8 ± 14.6	54.4 ± 14.9	51.2 ± 14.2	0.555
SOFA	10 (7, 13)	11 (7, 13)	9 (7, 12)	0.07
Charlson comorbidity index	5 (3, 6)	5 (3, 6)	4 (2, 6)	0.007
Mechanical ventilation, *n* (%)	459 (90.7%)	226 (91.5%)	233 (90%)	0.552

RRT = renal replacement therapy; CVVH = continuous venovenous hemofiltration; CVVHD = continuous venovenous hemodialysis; CVVHDF = continuous venovenous hemodiafiltration.

**Table 5:** Baseline characteristics for critically ill patients at 90-day mortality.

Variables	Total (*n* = 506)	Death (*n* = 268)	Live (*n* = 238)	*P* value
Age, years	64 (52, 74)	66 (54, 76)	61 (48.5, 70)	<0.001
Male, *n* (%)	306 (60.5%)	167 (62.3%)	139 (58.4%)	0.369
Heart rate, beats/min	106 (90, 120)	106 (90, 119)	104 (88, 123)	0.956
Mean arterial pressure, mmHg	56 (49, 63)	54 (47, 62)	57 (51, 64)	0.006
Respiratory rate, breath/min	28 (24, 34)	29 (24, 35)	28 (24, 33)	0.101
Creatinine admitted to ICU, mg/dL	2.9 (1.8, 4.4)	2.6 (1.7, 4.3)	3 (1.9, 4.6)	0.07
Creatinine in 24 hours before RRT initiation, mg/dL	4.2 (3, 5.5)	4 (2.9, 5.3)	4.3 (3.2, 5.9)	0.053
Duration of RRT (h)	105.8 (63.7, 189.8)	99.5 (63.8, 190.3)	110.4 (63.2, 184.3)	0.792
RRT modes, *n* (%)				
CVVH	14 (2.8%)	8 (3%)	6 (2.5%)	0.751
CVVHD	160 (31.6%)	93 (34.7%)	67 (28.2%)	0.114
CVVHDF	223 (44.1%)	98 (36.6%)	125 (52.5%)	<0.001
CVVH + CVVHD	88 (17.4%)	59 (22%)	29 (12.2%)	0.004
CVVH + CVVHDF	12 (2.4%)	6 (2.2%)	6 (2.5%)	0.835
CVVHD + CVVHDF	8 (1.5%)	3 (1.1%)	5 (2.1%)	0.484
Others	1 (0.2%)	1 (0.4%)	0	1
Main diagnosis class, *n* (%)				
Cardiovascular	105 (20.8%)	59 (22%)	46 (19.3%)	0.702
Trauma	63 (12.4%)	36 (13.5%)	27 (11.4%)	0.478
Respiratory	20 (4%)	10 (3.7%)	10 (4.2%)	0.786
Infection	124 (24.5%)	64 (23.9%)	60 (25.2%)	0.729
Others	194 (38.3%)	99 (36.9%)	95 (39.9%)	0.492
Sepsis, *n* (%)	95 (18.8%)	51 (19%)	44 (18.5%)	0.876
Chronic kidney disease, *n* (%)	176 (34.8%)	94 (35.1%)	82 (34.5%)	0.884
Simplified Acute Physiology Score II	52.8 ± 14.6	54 ± 15	51.4 ± 14.2	0.524
SOFA	10 (7, 13)	10 (6, 13)	9 (7, 12)	0.113
Charlson comorbidity index	5 (3, 6)	5 (3, 6)	4 (2, 6)	0.002
Mechanical ventilation, *n* (%)	459 (90.7%)	246 (91.8%)	213 (89.5%)	0.375

RRT = renal replacement therapy; CVVH = continuous venovenous hemofiltration; CVVHD = continuous venovenous hemodialysis; CVVHDF = continuous venovenous hemodiafiltration.

**Table 6:** The effect of the duration of RRT on mortality analyzed by Cox proportional hazards regression models.

Outcome	Unadjusted^*∗*^			Adjusted^*∗*^		
	HR	95% CI	*P* value	HR	95% CI	*P* value
*28-day mortality*						
Duration of RRT	0.996	0.994–0.998	<0.001	0.995	0.993–0.997	<0.001
*60-day mortality*						
Duration of RRT	0.999	0.998–1	0.055	0.999	0.998–1	0.052
*90-day mortality*						
Duration of RRT	0.999	0.999–1	0.207	0.999	0.998–1	0.06

RRT = renal replacement therapy. ^*∗*^Unadjusted: single-factor analysis of the effect of duration of RRT on mortality by Cox proportional hazards regression models. ^*∗*^Adjusted: adjusted by variables selected from the univariate analysis (*P* value less than 0.1).

## Data Availability

The data are available on the MIMIC-II website at https://archive.physionet.org/mimic2/.
